# Supplementary material for: A human antibody against Zika virus crosslinks the E protein to prevent infection
Source: Nat Commun. 2017 Mar 16;8:14722. doi: 10.1038/ncomms14722 (PMC5356071; doi:10.1038/ncomms14722)
Supplement: Supplementary Information — Supplementary Figures and Supplementary Table [file ncomms14722-s1.pdf]

Editorial Note: This manuscript has been previously reviewed at another journal that is not operating a transparent peer review scheme. This document only contains reviewer comments and rebuttal letters for versions considered at Nature Communications.

PEER REVIEW FILE

Reviewers' Comments:

Reviewer #1 (Remarks to the Author):

The revised version of the manuscript is much clearer than the initial version. However, this reviewer regrets that the comments provided as response 1e to the reviewer's is not also incorporated into the text; after all, is it to the reader that it is important to explain the additional insights provided in this paper from what had been initially described.

But overall, I feel that the manuscript is acceptable now for publication in Nature Communications.

RESPONSE TO REFEREE'S COMMENT:

Referee: ***The revised version of the manuscript is much clearer than the initial version. However, this reviewer regrets that the comments provided as response 1e to the reviewer's is not also incorporated into the text; after all, is it to the reader that it is important to explain the additional insights provided in this paper from what had been initially described. But overall, I feel that the manuscript is acceptable now for publication in Nature Communications.***

RESPONSE: We thank the referee for the kind comment. We have added a new paragraph to the manuscript from our rebuttal to point 1e. See page 8, lines 1-11 of the updated manuscript.
